# Investigation of Flame and Thermal Degradation Behavior of Xanthan- and Gelatin-Based Composites Used as Topsoil Covers in Forestry

**DOI:** 10.3390/molecules30163324

**Published:** 2025-08-08

**Authors:** Alessandro Sorze, Janine Bösing, Sebastian Hirschmüller, Andrea Dorigato

**Affiliations:** 1 Department of Industrial Engineering and INSTM Research Unit, University of Trento, 38123 Trento, Italy; 2 Department of Research, Development and Transfer, Technical University of Applied Sciences Rosenheim, 83024 Rosenheim, Germany; janine.boesing@th-rosenheim.de (J.B.);

**Keywords:** xanthan, gelatin, wood fibers, topsoil cover, flammability, thermal degradation, forestry

## Abstract

This study focused on investigating the flammability and thermal degradation behavior of wood fiber-reinforced composites consisting of xanthan gum (XG) and gelatin (GEL). These materials could potentially be used as novel bio-based and biodegradable topsoil covers (TSCs) to support reforestation practices. To improve the thermal properties of these composites, xanthan gum was cross-linked with citric acid (CA) or tannic acid (TA) and eventually coated with casein, while gelatin was cross-linked with tannic acid. Thermogravimetric analysis (TGA) showed that thermal degradation of all the prepared samples started at temperatures of 200 °C for xanthan-based samples and 300 °C for gelatin-based samples, which is well above the typical operating conditions for TSCs in their intended application. Single-flame-source tests demonstrated that the CA cross-linked xanthan-based TSCs coated with casein and all the gelatin-based TSCs had excellent self-extinguishing properties. Additionally, Limiting Oxygen Index (LOI) tests showed that gelatin-based composites had LOI values between 30 and 40 vol% O_2_, increasing with a higher gelatin-to-wood fiber ratio. These results demonstrated the potential of cross-linked biopolymers (e.g., xanthan and gelatin) as green flame retardants for the production of wood fiber-filled TSCs for use in forestry.

## 1. Introduction

An interesting approach supporting reforestation and promoting plant growth, especially under drought conditions, is using topsoil covers (TSCs). These multifunctional mulching films are designed to provide a more favorable environment for plant growth by increasing soil temperature [1,2], reducing water evaporation [3], and inhibiting the growth of competing weeds [4]. Due to their proven benefits, TSCs are becoming increasingly widespread in both forestry and agriculture [5,6,7,8,9,10]. The most common mulching products are plastic films [11,12], estimated to cover over 128,000 km^2^ of land worldwide [13]. Despite the economic benefits of these materials, the risk of microplastics being released into the soil through mechanical damage, UV degradation, or erosion by wind and rain is driving research into more environmentally friendly solutions [11,12,14,15]. Furthermore, the application of plastic films in the summer generally hinders plant growth due to the excessive increase in soil temperature and the inhibition of root growth caused by high heat stress [16]. To overcome these limitations, biodegradable solutions, such as starch, alginates, chitosan, proteins, and their combinations [17,18,19,20,21,22], have been investigated as an alternative. However, the market penetration of these products is still limited due to their low mechanical and water barrier properties, as well as their high production costs.

Based on these considerations, our research groups have recently developed two different types of bio-based and biodegradable composites that could be used as novel topsoil covers. The first type was based on xanthan gum (XG), a bio-based polysaccharide produced by *Xanthomonas campestris* under aerobic conditions using sugar cane, maize, or their derivatives as a source of carbohydrates [23,24,25,26]. Specifically, the composites were developed using XG cross-linked with either citric acid (CA) or tannic acid (TA) and reinforced with wood fibers. The second type of TSC was based on gelatin (GEL), a protein derived from animal waste containing collagen [27,28,29], cross-linked with tannic acid and reinforced with wood fibers. Adding CA and TA as cross-linkers to XG and GEL is fundamental to improving the thermal and mechanical properties of biopolymers, as well as enabling the formation of hydrogel structures that are insoluble in water. The specific formulations discussed in the present work are derived from preliminary experimental evaluations and optimizations reported in our previous papers [30,31,32,33,34,35]. These works demonstrated that xanthan-based TSCs cross-linked with CA or TA exhibited exceptional water absorption capabilities, even after multiple absorption/drying cycles (with an increase of up to 1900% over the original mass). Furthermore, samples cross-linked with CA displayed water vapor permeance values similar to those of commercial woven polypropylene (PP) mulching films and twice the penetration resistance of the commercial films. The presence of wood fibers in these TSCs provided dimensional stability and minimal shrinkage upon drying. Additionally, a planting trial experiment conducted in a nursery demonstrated that both types of TSCs had a positive impact on the growth rates of the above- and belowground plant parts and on soil microbial activity, enhancing bacteria richness and diversity. Outdoor weathering conditioning tests demonstrated that more than 60% of the sample mass was biodegraded after 7 months of exposure.

Considering that the purpose of these TSCs is to support plant growth in forestry applications, and they are mainly constituted of biopolymers and wood fibers, a primary concern may be the risk of flammability of these products and the subsequent development of forest fires. According to the annual report of the EFFIS (European Forest Fire Information System), due to climate change, almost 900,000 hectares of land burnt in the EU in 2022, compared to an average of 349,000 hectares between 2006 and 2023 [36]. Furthermore, an average of 2.8 million hectares burnt each year in the US during the same period, according to the National Interagency Fire Center [37]. The damage caused by wildfires is problematic not only in the short term, but also in the long term. For example, wildfires are often associated with increased soil erosion, altering soil morphology and resulting in changes to porosity, increased bulk density, and reduced aggregate stability [38,39,40]. Fires are also associated with nutrient depletion, increased rainwater runoff, and water repellency [41,42,43]. Furthermore, they hinder tree regeneration and reduce forest resilience, potentially displacing resilient forests, such as coniferous ones, with shrub or grassland ecosystems [44,45,46].

Recent research into flame retardants has focused on the use of biopolymers and other sustainable alternatives. Specifically, natural polymers such as chitosan, gelatin, starch, alginate, and lignin are being investigated for their inherent carbon content and ability to form protective char layers upon thermal decomposition [47,48,49,50,51]. Therefore, a systematic investigation of the flammability conditions of the biopolymer-based TSCs previously developed by our research groups is fundamental in view of the possible application of these materials in forestry.

The main objective of this study was thus to investigate the flammability and thermal degradation of novel xanthan- and gelatin-based wood fiber composites. This ensured that, if applied as TSCs in forestry, these materials would not act as a fire accelerant for forest fires. To the best of our knowledge, this is the first study investigating the flame response and thermal behavior of xanthan- and gelatin-based TSCs.

## 2. Results and Discussion

### 2.1. Fourier-Transformed Infrared (FT-IR) Spectroscopy

**Figure 1:**
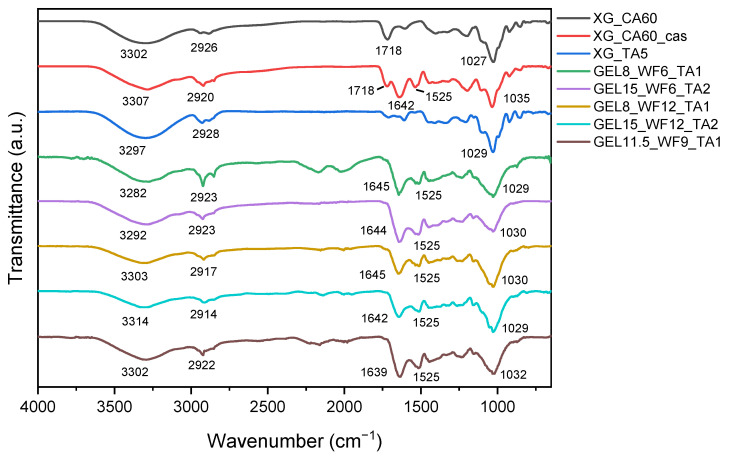
Normalized FT-IR spectra of the prepared xanthan- and gelatin-based wood fiber composite TSCs.

Figure 1 shows the normalized FT-IR spectra of TSC samples, which were used to investigate the chemical structure of the materials.

**Figure 2:**
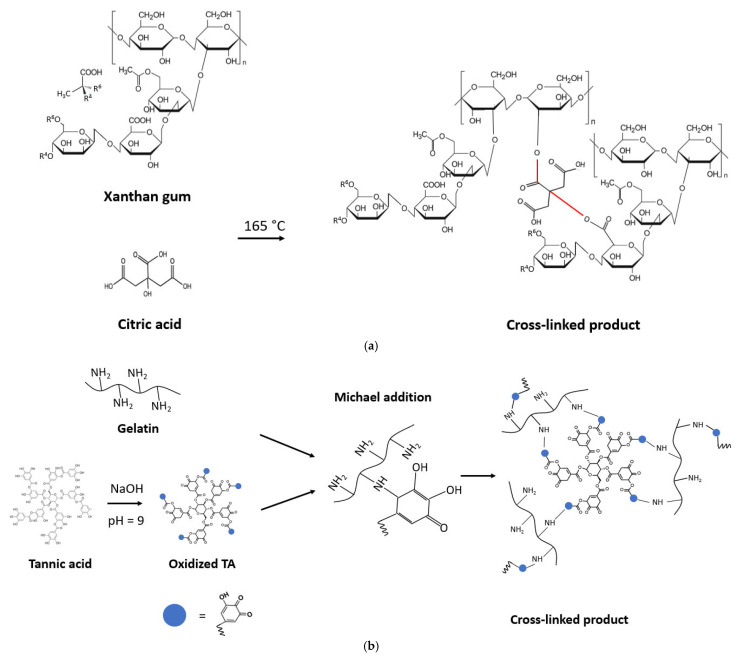
Cross-linking reaction scheme of (**a**) xanthan gum with citric acid and (**b**) gelatin with tannic acid.

Figure 1 shows a broad absorption peak for all samples at around 3300 cm^−1^, which corresponds to the O-H stretching vibration of the hydroxyl groups in biopolymers. The peaks at around 2920 cm^−1^ can be assigned to the CH groups in xanthan, gelatin, and wood fibers. The samples XG_CA60 and XG_CA60_cas exhibit a peak at 1718 cm^−1^ due to the presence of ester groups resulting from the esterification reaction between xanthan gum and wood fibers with the citric acid cross-linking agent [33,52]. For xanthan gum, this reaction occurs between the R-OOH group in the D-glucuronic unit of XG and the only R-OH group in CA, as schematized in Figure 2a. For wood fibers, the reaction occurs between the hydroxyl groups of cellulose and the carboxyl groups in CA [53,54,55]. Peaks at around 1642 and 1510 cm^−1^ are clearly visible in all gelatin-based TSCs and in XG_CA60_cas. These peaks are associated with the Amide-I (C=O stretching) and the Amide-II (N-H bending) functional groups present in both gelatin and casein [56,57,58,59]. Finally, for all the samples, the peaks between 1027 and 1032 cm^−1^ can be associated with the stretching vibration of C-O present in xanthan, gelatin, and wood fibers. For the TA cross-linked gelatin samples, the very broad peak at 1030 cm^−1^ could also be related to the superposition of another signal, namely the C–H vibration of the benzene ring near the phenol –OH groups of TA [60]. Indeed, the cross-linking reaction between gelatin and TA occurs through a one-step Michael addition reaction under oxidizing conditions in an alkaline environment (Figure 2b). Taking these considerations into account, and bearing in mind that the samples are insoluble in water (as observed in the author’s previous work [30,31,32,33,34,35]), it is possible to assess the correct sample preparation, and that the cross-linking reactions between the biopolymers and their respective cross-linking agents have correctly occurred.

### 2.2. Thermogravimetric Analysis (TGA)

**Figure 3:**
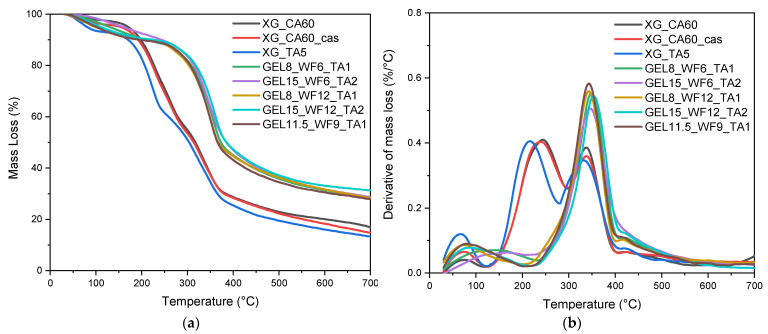
(**a**) TGA and (**b**) DTG curves of the prepared TSC samples.

TGA is an important technique for evaluating the thermal stability and decomposition of materials. Specifically, Figure 3a,b and Table 1 report the most important results of the TGA, i.e., the temperature at which degradation begins (T_onset_), the degradation temperature (T_d_), considered as the maximum of the first derivative of the TGA thermogram (DTG), and the residual mass at 700 °C (m_R,700_).

Figure 3a shows an initial mass loss of around 7% for all the samples between 35 and 125 °C, which is associated with moisture evaporation. As reported in Table 1, the first significant mass loss (approximately 30%) for the xanthan-based TSCs occurs between 220 and 245 °C (T_d,1_), associated with glycerin degradation [61]. The degradation of XG and the wood fibers occurs between 330 and 340 °C (T_d,2_) with two superimposed peaks due to the chemical similarities of these constituents [62]. The casein coating does not significantly affect the TGA results of the CA-cross-linked samples. For the gelatin-based TSC samples, only one degradation peak is observed, between 347 and 360 °C (T_d,2_), which is associated with the combined degradation of gelatin, wood fibers, and tannic acid [63]. The residual mass of the gelatin samples at 700 °C is almost twice that of the xanthan samples, demonstrating the formation of a stable char layer due to the cross-linking between gelatin and TA, i.e., the formation of a thermally stable, three-dimensional network that is more resistant to thermal decomposition [63,64]. On a general level, it can be concluded that the decomposition temperature of all the prepared materials is well above their operating conditions and comparable to that of biopolymer-based composites found in the literature [65,66,67].

### 2.3. Single-Flame-Source Tests

**Figure 4:**
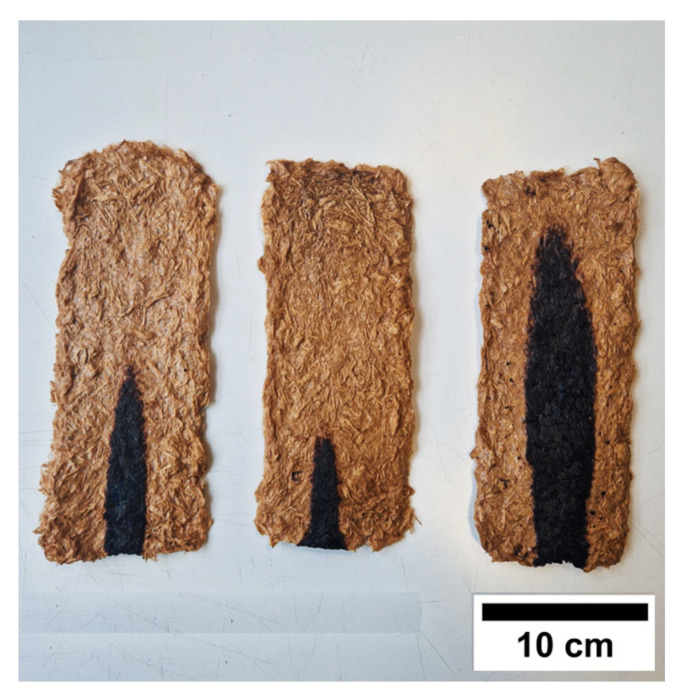
Representative xanthan-based TSC specimens (XG_CA60) after single-flame-source tests.

Figure 4 shows three representative images of xanthan-based TSCs after the single-flame-source tests, while Table 2 summarizes the main results obtained from these samples.

The results of the single-flame-source test of the xanthan-based TSCs reveal different behaviors. The two samples cross-linked with citric acid did not burn, and the flame self-extinguished a few seconds after the burner had been removed from the samples. As shown in Figure 4 and Table 2, the presence of the casein coating on the TSC surface slightly reduces the burnt area. Additionally, the presence of casein significantly limits mass loss due to combustion, highlighting its promising behavior as a green flame retardant. As reported in the literature, casein is characterized by high phosphorus and nitrogen contents and has been proven to be a potential bio-based flame retardant additive [68,69]. No flaming droplets could be observed in either sample, though white smoke developed for a few seconds after the flame was extinguished. Conversely, specimens cross-linked with tannic acid (composition XG_TA5) failed the test because the flame propagated along the specimens for more than 150 mm, exceeding the standard specification. This different behavior compared to the CA cross-linker could be explained by the different cross-linking mechanisms involving XG and wood fibers. As the FT-IR analysis showed, xanthan gum and wood fibers react with citric acid through esterification reactions. These covalent ester bonds are thermally stable and reduce the flammability of the samples. Furthermore, research studies have shown that citric acid forms a stable char layer upon combustion of cellulose, which helps to limit flame propagation [70,71]. Conversely, tannic acid may release flammable volatiles when heated, which could have a negative impact on the flame retardant properties of the materials. However, there is currently no research on the cross-linking effects of tannic acid on xanthan gum.

None of the gelatin-based TSC compositions burned and the flame self-extinguished as soon as the burner had been removed from the sample. No flaming droplets could be observed for these samples; instead, white smoke developed for a few seconds after the flame was extinguished.

**Figure 5:**
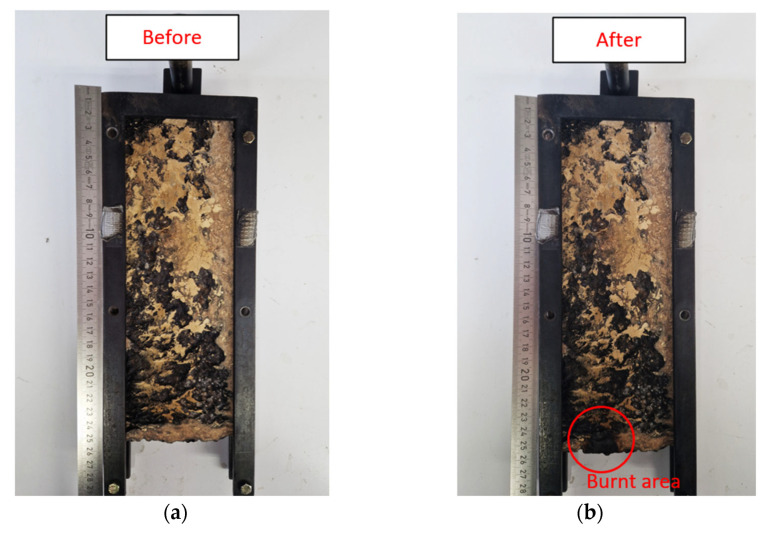
Representative image of a gelatin-based specimen (GEL8_WF6_TA1): (**a**) before and (**b**) after the single-flame-source test.

Figure 5a,b shows a representative image of a gelatin-based specimen (GEL8_WF6_TA1) before and after the single-flame-source test.

As can be seen in Figure 5b, the burnt area is very limited. Specifically, the burnt height reaches a maximum of 20 mm from the base of the sample for all the compositions. The high nitrogen content of amino acids in gelatin suggests that it is a low-flammability material [48,72,73]. In this case, the presence of tannic acid does not negatively impact the flammability properties because it forms strong bonds with proteins, such as gelatin, reducing the release of volatiles during combustion [63,74]. For all of these compositions, the burnt area of the samples expanded in volume. This behavior is specific to gelatin, which expands and releases gases (mainly CO_2_ and NH_3_) during combustion, thus promoting flame extinction [75]. These non-flammable gases displace the oxygen in the flame zone, reducing the local concentration below the combustion threshold. In addition, the volume expansion due to gas evolution leads to intumescent behavior. This results in the formation of a swollen, foamed char layer that prevents heat release and volatile formation during combustion, thereby inhibiting further burning [64].

### 2.4. Limiting Oxygen Index (LOI)

**Figure 6:**
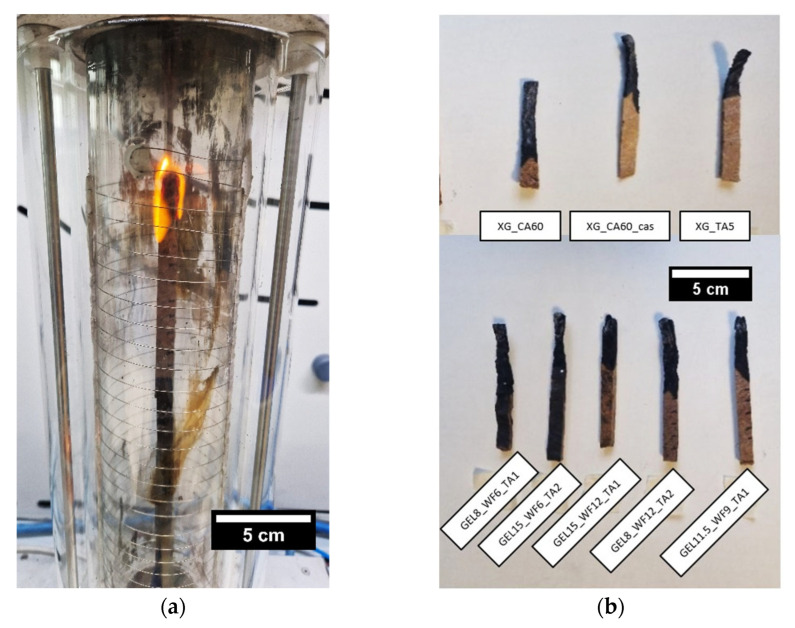
Representative images of (**a**) GEL8_WF12_TA1 TSC sample during the LOI test, and (**b**) of the tested specimens.

LOI tests were carried out in order to measure the minimum oxygen concentration, expressed as a percentage by volume, at which the material can sustain flame combustion in a mixture of oxygen and nitrogen gas. Figure 6a shows a representative picture of a TSC specimen (GEL8_WF12_TA1) during the LOI test, Figure 6b shows images of the tested specimens, and Table 3 reports the LOI values for the prepared TSC samples.

As shown in Table 3, the xanthan-based TSCs cross-linked with citric acid (XG_CA60 and XG_CA60_cas) have a LOI value higher than the standard oxygen condition in air (20.95%). The addition of a casein coating to the surface of the XG-based samples does not significantly affect this result. However, it should be considered that the casein coating is a surface treatment, and the LOI test requires applying a flame to the edge of the specimen, which was not coated with the casein layer. Conversely, the composition with tannic acid shows LOI values very close to the oxygen concentration in air, confirming limited flame retardant properties.

Gelatin-based TSCs have higher LOI values, confirming their ability to resist combustion in air. Indeed, the LOI values correlate to the sample composition. Specifically, increasing the ratio of gelatin to wood fibers results in a higher LOI, while compositions with a lower relative gelatin content (i.e., GEL8_WF12_TA1) have lower LOI values. These results further demonstrate the capability of gelatin as a flame retardant. The LOI values obtained for the gelatin-based samples are comparable to those reported in the literature for other fire retardant-coated wood samples. [76,77]. Moreover, these values are higher than critical oxygen thresholds for the combustion of commercial synthetic polymers and can be compared with the values of other self-extinguishing and non-flammable polymers like polytetrafluoroethylene (PTFE) and polyvinylidene fluoride (PVDF) [78,79].

## 3. Materials and Methods

### 3.1. Materials

Commercial-grade amorphous xanthan gum (XG) with a molecular weight (MW) of 1 × 10^6^ g/mol and purity higher than 91% was supplied by Galeno Srl (Prato, Italy) in the form of a fine powder. GELITA IMAGEL^®^ LB, a type-B semicrystalline gelatin powder with a bloom value of 113 (gel) and viscosity of 2.29 mPa∙s at 6.67% gelatin content and 60 °C solution temperature, was provided by GELITA AG (Eberbach, Germany). STEICO^®^ Flex-036 wood fibers, with a bulk density of 60 kg/m^3^ and an aspect ratio (length to diameter ratio) ranging from 22.5 to 75 mm/mm, were purchased from STEICO SE (Feldkirchen, Germany). Vegetable glycerin, which was used as a plasticizing agent, with a MW of 92.1 g/mol and purity greater than 98%, was supplied by Farmalabor srl (Assago, Italy). Citric acid monohydrate (CA) with a MW of 210.1 g/mol and purity of 99.5%, used as a cross-linking agent, was purchased from Riedel-de Haën GmbH (Seelze, Germany). Tannic acid chestnut powder (TA), which was also used as a cross-linking agent, with a MW of 1701.2 g/mol, was provided by W. Ulrich GmbH (Eresing, Germany). Casein, used in solution as a protective coating, with a MW of 23,700 g/mol, was obtained by Thermo Fisher Scientific Inc (Waltham, MA, USA). Sodium hydroxide (NaOH) microbeads, used as a pH adjustment, were supplied by WHC GmbH (Schweitenkirchen, Germany). All materials were used as received.

### 3.2. Sample Preparation

**Figure 7:**
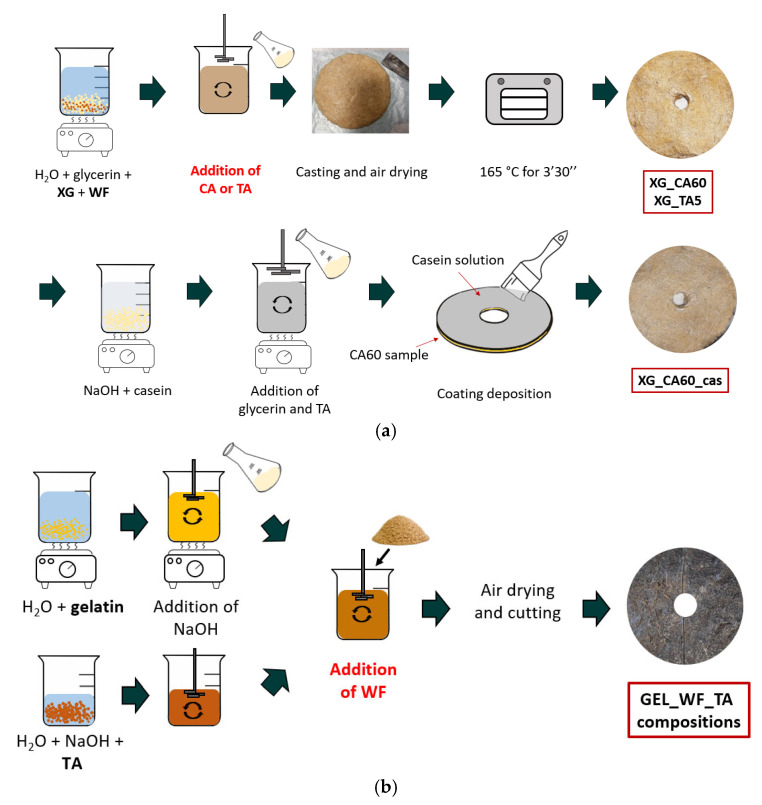
Sample preparation scheme of (**a**) xanthan-based TSCs and (**b**) gelatin-based TSCs. XG: xanthan gum; WF: wood fibers; CA: citric acid; TA: tannic acid.

The production process for xanthan-based TSCs is thoroughly described in [33,34] and schematized in Figure 7a. XG, wood fibers, and glycerin (with relative ratios reported in Table 4) were mixed in hot water (60 °C) until homogeneity was reached (5 min). Then, citric acid (60 wt% relative to the XG amount) or tannic acid (5 wt% relative to the XG content) was added as a cross-linking agent to produce two different compositions (XG_CA60 and XG_TA5). These specific formulations and material ratios were selected because they showed the most promising results in terms of water absorption, water vapor permeability, and resistance to perforation [33]. The samples were dried under controlled conditions (T = 24 °C, RH% = 40%) for 2 days, then thermally treated in an oven at 165 °C for 3.5 min to activate the cross-linking reactions between xanthan gum, wood fibers, and the cross-linking agents [54]. A third sample (XG_CA60_cas) was produced by applying a top-side protective layer of an alkaline solution of casein to sample XG_CA60. The casein coating was prepared according to the methodology described by Picchio et al. [80]. Specifically, casein was dissolved at 40 °C in an alkaline solution (1 M NaOH) under magnetic stirring, glycerin (50 wt% of casein) and tannic acid (10 wt% of casein) were then added. Before use, the solution was stirred for 1 h.

The gelatin-based composites were prepared according to the procedure already described in [34] and schematized in Figure 7b. Gelatin was mixed in water at 55 °C for 120 min until the gelatin powder was completely dissolved. During the mixing process, the pH of the solution was adjusted to nine by gradually adding NaOH. In parallel, tannic acid was dissolved in a small amount of water at room temperature, and the pH was adjusted in the same way as for the gelatin solution. Subsequently to the pH adjustment, both solutions were mixed under permanent stirring, and the wood fibers were added. The homogeneous solution was then poured into a mold and cured under controlled airflow conditions (T = 24 °C, RH% = 40%) for two weeks. Initially, by changing the ratios of gelatin, tannic acid, wood fibers, and water, nine different compositions were produced according to a face-centered central composite design (FCCD), with eight corner points and one center point (CP). Preliminary evaluations revealed that only five out of nine compositions should be studied in this work, as reported in Table 5.

### 3.3. Experimental Techniques

#### 3.3.1. FT-IR Spectroscopy

The tests were conducted in attenuated total reflectance (ATR) mode using a PerkinElmer Spectrum One spectrometer (PerkinElmer, Waltham, MA, USA), in the wavelength number range of 650–4000 cm^−1^. Each spectrum was obtained from the superposition of four replicates.

#### 3.3.2. Thermogravimetric Analysis (TGA)

TGA was performed using a thermobalance Mettler TG 50 (Mettler Toledo Inc., Columbus, OH, USA). Approximately 20 mg of a specimen was subjected to a thermal ramp between 30 °C and 700 °C at a heating rate of 10 °C/min under a constant nitrogen flow of 10 mL/min. The test was performed on 3 replicates for each sample.

#### 3.3.3. Single-Flame-Source Tests

**Figure 8:**
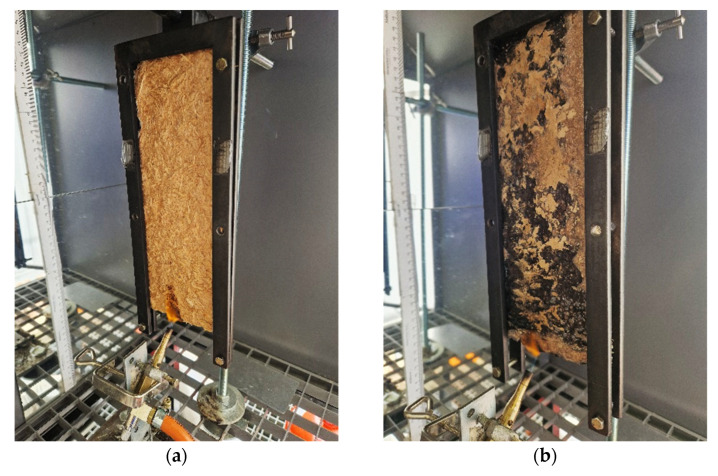
Representative images during the single-flame-source test on TSCs (**a**) XG_CA60 and (**b**) GEL8_WF6_TA1 samples.

Single-flame-source tests were carried out based on the EN ISO 11925-2 standard. Vertically clamped specimens with dimensions of 250 mm (vertical length) by 90 mm (horizontal width) and 10 mm (thickness) were put in contact with a 20 mm high flame on the bottom edge for 30 s. Figure 8a,b shows two representative images of TSC samples during the single-flame-source test. After removing the burner, the time until ignition and the time until the flame tip reached 150 mm in height above the point of flame impingement were recorded. The test was performed on 5 replicates for each sample.

#### 3.3.4. Limiting Oxygen Index (LOI)

Measurements were performed according to ASTM D2863 by using a CEAST oxygen index apparatus (CEAST SpA, Torino, Italy) on at least ten replicates of each composition with the dimensions 100 mm × 10 mm × 10 mm.

#### 3.3.5. Statistical Analysis

All the results have been presented as the mean ± standard error of the mean. The data of single-flame-source tests and LOI analyses were analyzed by using one-way analysis of variance (ANOVA) with a significance level of 0.05. Pairwise differences between treatments were assessed using the post hoc Tukey’s test.

## 4. Conclusions

In this work, the flammability and thermal degradation behavior of novel bio-based and biodegradable topsoil covers, based on two different biopolymers (xanthan gum or gelatin) and reinforced with wood fibers, were investigated. To improve thermal stability, xanthan gum was cross-linked with citric acid or tannic acid and, then, eventually coated with casein, while gelatin was cross-linked with tannic acid. FT-IR analysis demonstrated the effective occurrence of the cross-linking reactions within the prepared TSCs. TGA results showed that the xanthan gum-based samples started to thermally degrade at around 200 °C and the gelatin-based TSCs at around 300 °C, well above the typical working conditions of TSCs for the intended application. Single-flame-source tests revealed the flame self-extinguishing capability of both CA cross-linked xanthan-based TSCs and all the gelatin-based TSCs. Furthermore, gelatin-based samples showed higher LOI values (up to 41 vol% O_2_), particularly when the gelatin-to-wood fiber ratio was increased. Thus, it could be concluded that TSC composites produced from CA-cross-linked xanthan gum or TA-cross-linked gelatin and reinforced with wood fibers could safely be used for reforestation practices without introducing a potential ignition source for wildfires. A possible limiting factor for the upscaling of these materials could be the high cost of biopolymers and the energy-intensive production processes required for these composites. However, the continuous increase in biopolymer production in recent years is expected to significantly improve the economic applicability of the proposed system. Future steps will focus on optimizing production processes to reduce energy consumption and costs, thereby making these products easily scalable.

## Figures and Tables

**Table 1:** Results of the TGA on the prepared TSCs.

Sample	T_onset_ (°C)	T_d,1_ (°C)	T_d,2_ (°C)	m_R,700_ (%)
XG_CA60	152.7	243.0	338.2	16.8 ± 1.5
XG_CA60_cas	160.1	239.5	337.8	14.8 ± 2.1
XG_TA5	153.8	220.1	332.2	13.2 ± 1.1
GEL8_WF6_TA1	255.8	-	356.5	28.4 ± 3.2
GEL15_WF6_TA2	248.3	-	347.5	28.7 ± 1.1
GEL8_WF12_TA1	250.1	-	351.0	28.3 ± 0.8
GEL15_WF12_TA2	256.3	-	360.2	31.3 ± 2.5
GEL11.5_WF9_TA1	249.8	-	350.5	27.9 ± 1.8

T_onset_: temperature at which degradation begins; T_d,1_: temperature of first degradation; T_d,2_: temperature of second degradation; m_R,700_: residual mass at 700 °C.

**Table 2:** Results of the single-flame-source tests on xanthan-based samples.

Sample	Mass Loss (%)	Burnt Height (cm)	Burnt Width (cm)	Combustion *
XG_CA60	1.6 ± 0.1 ^a^	3.0 ± 0.6 ^ab^	9.7 ±2.2 ^a^	no
XG_CA60_cas	0.3 ± 0.1 ^b^	2.4 ± 0.5 ^a^	7.0 ± 1.9 ^a^	yes
XG_TA5	9.5 ± 0.2 ^c^	3.8 ± 0.8 ^b^	13.3 ± 5.6 ^a^	yes

* The flame tip reached 150 mm above the point of application of the flame, according to EN ISO 11925-2. Different letters in a column indicate that results are statistically different (*p* < 0.05).

**Table 3:** LOI values of the prepared TSCs.

Sample	LOI (vol% O_2_)
XG_CA60	23.5 ± 0.1 ^a^
XG_CA60_cas	23.7 ± 0.1 ^a^
XG_TA5	22.4 ± 0.2 ^a^
GEL8_WF6_TA1	36.3 ± 0.1 ^bc^
GEL15_WF6_TA2	41.5 ± 0.1 ^c^
GEL8_WF12_TA1	31.2 ± 0.2 ^ab^
GEL15_WF12_TA2	33.6 ± 0.1 ^abc^
GEL11.5_WF9_TA1	33.3 ± 0.1 ^abc^

Different letters in a column indicate that results are statistically different (*p* < 0.05).

**Table 4:** Composition of the prepared XG/wood fiber-based samples before drying (i.e., in the wet state).

Sample	Xanthan Gum (wt%)	Wood Fibers (wt%)	Glycerin (wt%)	CA (wt%)	TA (wt%)	Water (wt%)	Casein Coating
XG_CA60	3.5	3.5	4.2	2.0	-	86.8	no
XG_CA60_cas	3.5	3.5	4.2	2.0	-	86.8	yes
XG_TA5	3.5	3.5	4.2	-	0.2	88.6	no

CA: citric acid; TA: tannic acid.

**Table 5:** Composition of the prepared gelatin/wood fiber-based samples before drying (i.e., in the wet state).

Sample	Gelatin (wt%)	Wood Fibers (wt%)	TA (wt%)	Water (wt%)
GEL8_WF6_TA1	8.0	6.0	1.0	85.0
GEL15_WF6_TA2	15.0	6.0	2.0	77.0
GEL8_WF12_TA1	8.0	12.0	1.0	79.0
GEL15_WF12_TA2	15.0	12.0	2.0	71.0
GEL11.5_WF9_TA1	11.5	9.0	1.0	78.5

TA: tannic acid.

## Data Availability

The raw data supporting the conclusions of this article will be made available by the authors on request.

## Abbreviations

The following abbreviations are used in this manuscript:

TSC	Topsoil cover
XG	Xanthan gum
CA	Citric acid
TA	Tannic acid
GEL	Gelatin
WF	Wood fibers
FT-IR	Fourier-transformed infrared spectroscopy
TGA	Thermogravimetric analysis
LOI	Limiting oxygen Index
